# Modern research of TCM etiology and pathogenesis theory and translational medicine

**DOI:** 10.1186/1479-5876-10-S2-A36

**Published:** 2012-10-17

**Authors:** Ping Liu

**Affiliations:** 1 Shanghai University of Traditional Chinese Medicine, Shanghai, 201203, China

## 

To develop the basic theory based on clinical practice, and further to guide clinical for improving the therapeutic effect is one of the important characteristics of TCM thinking way. In a sense, the development and innovation of TCM basic theory is equivalently a process of translational medicine. The typical examples are “defense, qi, nutrient and blood pattern identification” and “triple energizer pattern identification”.

The knowledge and technology using in modern integration of TCM syndrome and western medicine disease are always as a starting point to solve the clinical problems, to improve the therapeutic efficacy of the disease and the patient's quality of life. The methods of TCM are to establish a series of systematic research from the clinical (efficacy) – experiment – drug efficacy mechanism, and constantly to find and solve new problems, thereby to promote the development of medicine.

TCM etiology and pathogenesis theory is based on the visceral manifestation, meridian and collateral, essence, Qi, blood, fluid and humor, etc. Judging by clinical observation, it is eventually verified whether the treatment effect can be overall restored. Against pathological features of modern diseases, TCM has given full play to the advantage of macro overall thinking, cognized and found TCM basic etiology and pathogenesis of the disease or the critical key affecting disease development and prognosis. To explore TCM effective therapies and formulas, further to develop TCM etiology and pathogenesis theory for understanding deeply western diseases, is essentially a kinds of the crossover and recreation process between TCM and western medicine.

The etiology and pathogenesis theory belongs to the “principles” scope within TCM “principles, methods, formulas and medicinals”. It must be verificated repeatly which combines closely with the therapeutic effects. Then it could form the etiology and pathogenesis theory which guides widely the diagnosis and treatment of clinical disease. That also is a key issue of TCM possessing "translational medicine" features.

In order to adapt to the development of modern society, and promote the theory innovation, TCM etiology and pathogenesis theory must adhere to the own theory and thinking principles, connect closely with the development of modern science, explore more convincing scientific evidence, and be constantly revised and improved in practice. Thereby it will fully show the practical significance in improving the clinical efficacy. At the same time, it is also an important way that finds a new prescription or a new use of mature prescription on treatment modern complex, refractory disease.

We have once analyzed the patients with post-hepatitis B cirrhosis under the guidance of traditional Chinese medicine thinking, which conjecture the disease reasons based on ZHENG and verify them according to clinical efficacy. Through clinical practice and large samples epidemiological survey combined with analytical processing technology, we found that “Qi Deficiency and Blood Stasis” is the basic DISEASE pathogenesis of liver cirrhosis, “Liver and Kidney Yin Deficiency” and “Dampness-Heat Smoldering” are the major ZHENG pathogenesis of liver cirrhosis.

Targeted to liver cirrhosis disease and ZHENG pathogenesis, we have adopted 4 classical prescriptions with different effects, such as “boosting qi”, “tonifying yin”, “eliminating stasis” and “clearing heat and draining dampness”, to carry out the experiments in 4 different classic animal models of liver cirrhosis. We found that Huangqi decoction could have good therapeutic effect within three models of liver cirrhosis. The results provided the experimental basis for establishing the liver cirrhosis TCM pathogenesis theory of “deficiency and detriment generating the accumulation”. Furthermore, using liver histology assessment as the primary efficacy endpoint, we found that Huangqi decoction could improve liver histopathological status of cirrhosis, compared with Fuzheng Huayu decoction, which can reinforce the healthy qi and resolve stasis. Consequently, we confirmed that “boosting qi and engendering essence, tonifying and replenishing to improve the deficiency and the detriment” are the fundamental principle on treating liver cirrhosis.

According to the active mechanism of tonifying and replenishing medicinal, combined with TCM essential and qi mutual transformation theory, we analyzed the pathobiological basis of liver cirrhosis TCM pathogenesis theory of “deficiency and detriment generating the accumulation”. The major characteristics of tonifying and replenishing medicinal on treating liver cirrhosis could include inhibiting the activation of hepatic stellate cell and liver epithelial-mesenchymal transition, inhibiting hepatocyte apoptosis, protecting liver sinusoidal endothelial cells and hepatic parenchymal cells. The outcomes communicated the concepts of TCM pathogenesis and modern pathobiology, and systematically demonstrated the connotation of liver cirrhosis TCM pathogenesis theory of “deficiency and detriment generating the accumulation” and treating disease from the root.

